# The medical transition of young adults with type 1 diabetes (T1D): a retrospective chart review identifies areas in need of improvement

**DOI:** 10.1186/s13633-020-00080-8

**Published:** 2020-05-28

**Authors:** Abby M. Walch, Carmen E. Cobb, Shirng-Wern Tsaih, Susanne M. Cabrera

**Affiliations:** 1 Department of Pediatrics, Medical College of Wisconsin, 8701 Watertown Plank Rd, Wauwatosa, WI 53226 USA; 2 Department of Obstetrics and Gynecology, Medical College of Wisconsin, 8701 Watertown Plank Rd, Wauwatosa, WI 53226 USA

**Keywords:** Type 1 diabetes, Transition, Adolescence, Hypertension, Dyslipidemia, Microalbuminuria, Retinopathy, Neuropathy

## Abstract

**Background:**

The transition process from pediatric to adult care in individuals with T1D has long-term ramifications on health outcomes. Recognition of differences in care delivery and changes made in management during this time may improve the process. We hypothesized that pediatric providers would be less likely to address T1D-related comorbidities than their adult counterparts, highlighting opportunities to strengthen care.

**Methods:**

A retrospective chart review of patients aged 16–21 years diagnosed with T1D before age 18 was performed. Data on diagnosis, screening, and management of hypertension, dyslipidemia, microalbuminuria, retinopathy, and neuropathy were collected for 1 year before and 1 year after transition to adult care. The ‘ADA Standards of Medical Care in Diabetes’ were used to determine adherence to the above parameters. Data before and after transition was compared by Fischer’s Exact and Exact McNemar tests.

**Results:**

Complete medical records for 54 subjects were reviewed before and after transition from pediatric to adult care providers within a single academic medical system (52% male; 78% Caucasian). Transition to adult care occurred at a mean age of 18 years. Mean length of transition was 7.8 months with no significant change in an individual’s HbA1c over that time. Over the transition period, there was no difference in diagnoses of hypertension or the use of anti-hypertensive. Adherence to lipid and retinopathy screening was similar across the transition period; however, adherence to microalbuminuria screening was higher after the transition to adult providers (*p* = 0.01). Neuropathy screening adherence was overall poor but also improved after transition (*p* < 0.001).

**Conclusions:**

Overall, there were no significant changes in the diagnosis or management of several T1D-related comorbidities during the transition period in a small cohort of young adults with T1D. The transition length was longer than the recommended 3-months, highlighting an opportunity to improve the process. There was no deterioration of glycemic control over this time, although HbA1c values were above target. Adult providers had significantly higher rates of adherence to screening for microalbuminuria and neuropathy than their pediatric counterparts, but adherence for neuropathy was quite poor overall, indicating a need for practice improvement.

## Background

The transition from pediatric to adult care for patients with T1D is a critical time when patients establish lifelong patterns of behavior and assume more responsibility for their diabetes self-management. Successes or failures during this transition have implications for the incidence of both acute and chronic complications [1]. Young adulthood is in general a time of poor glycemic control [2–4]. Most individuals fail to achieve the glycemic targets known to reduce the risk of chronic T1D complications [3, 5–8]. Further, prior studies have demonstrated worsening glycemic control during the transition from pediatric to adult care in patients with T1D, making this a particularly high-risk period [9]. Suboptimal glycemic control has been associated with poor health outcomes including the development of hypertension [10], increased mortality (all-cause and cardiovascular), and ischemic heart disease [11]. Furthermore, the presence of any one diabetes-associated complication has been shown to be associated with a higher risk of developing additional complications [11, 12]. Unfortunately, individuals diagnosed with T1D in childhood are at increased risk for developing associated microvascular and macrovascular complications later in life, and this risk increases with longer duration of diabetes [11–14]. Indeed, nearly a third of young adults diagnosed with T1D before age 20 in the United States have evidence of a T1D-related complication or comorbidity [15]. Despite this heightened risk, studies have shown decreased rates of screening for complications during young adulthood [16]. The American Diabetes Association (ADA) provides clear guidelines for screening and treatment of T1D-associated complications and comorbidities through its yearly release of ‘Standards of Medical Care in Diabetes’ [17–19].

To make the transition from pediatric to adult care more seamless and to improve long-term health outcomes for patients with T1D, further investigation of the changes made to the diabetes care these patients receive before and after transition to adult care is needed. If gaps in care are identified, they should ideally be addressed and incorporated into anticipatory care by pediatric providers. Anticipatory guidance has long been a central tenet of pediatric care in that it sets expectations and improves health; in fact, anticipatory guidance around diabetes-related topics in young adults with T1D has been associated with higher satisfaction with healthcare and overall health [20]. Additionally, patient education may offer promise in achieving target glycemic control in order to prevent complications [1]. Education efforts should be directed to the patient as they gradually take on increasing responsibility of their cares, transitioning away from parental supervision [1]. Transition programs that incorporate intensive patient education curricula serve to improve adherence to screening for T1D-related comorbidities as well as post-transition metabolic outcomes [21, 22].

This retrospective chart review specifically focuses on the screening, diagnosis, and management of several important T1D-related comorbidities in a cohort of young adults receiving pediatric and adult care in an academic medical center. The comorbidities of hypertension, dyslipidemia, microalbuminuria, retinopathy, and neuropathy were selected for study as these are known to have long-term ramifications on health outcomes and are generally assumed to affect adults more than children. We therefore hypothesized that pediatric providers would be less adherent than adult providers to the recommended screening and treatment for these comorbidities. If so, this study would provide an opportunity for practice improvement.

## Methods

After obtaining approval from the Children’s Hospital of Wisconsin and Froedtert/Medical College of Wisconsin Institutional Review Boards, a retrospective chart review was performed to investigate changes in the screening, diagnosis, and management of T1D-related co-morbidities during the transition phase between pediatric and adult providers. Subjects aged 16–21 years with at least two outpatient clinic visits to the Children’s Hospital of Wisconsin (CW) Diabetes Clinic between 7/31/2004 to 2/15/2018 were identified by searching the electronic medical record with the ICD-9 code specific for T1D (250.01). Subjects were included in further analysis if they received the diagnosis of T1D prior to age 18, initially received their care in the CW Diabetes Clinic, and subsequently transitioned their care to the adult practice at Froedtert & the Medical College of Wisconsin (MCW) Diabetes Clinic. Children’s Hospital of Wisconsin and Froedtert are institutions affiliated with the MCW academic medical center but have distinct clinical practices. The full electronic medical records were available for review in the pediatric and adult clinics. Subjects were excluded if they were determined to not have T1D, were lost to follow-up during the transition phase, or did not transition their adult care to MCW as records were not available for detailed review.

Data collected on each subject included gender, ethnicity, age at diagnosis, age at transition, and length of transition (i.e. number of days between the last pediatric clinic visit and the first adult clinic visit). Data on diagnosis, screening, and management of hypertension, dyslipidemia, microalbuminuria, retinopathy, and neuropathy was collected for 1 year prior to and 1 year after transition to adult care for each subject. The American Diabetes Association ‘Standards of Medical Care in Diabetes – 2018’ were used to determine adherence to the above parameters [17–19]. Additional data collected included the patient’s last recorded BMI (z-score) at a pediatric clinic visit and their hemoglobin A1c (HbA1c) level from the last pediatric clinic visit and from the first adult clinic visit. HbA1c levels at the last pediatric clinic visit of subjects included in the study were also compared to HbA1c levels of subjects excluded from the study because they transferred care to adult providers outside the MCW adult practice. HbA1c values were obtained by DCA Vantage Analyzer point-of-care system (Siemens Healthineers, Malvern, PA, USA) that has a maximum quantified HbA1c value of 14%; therefore, HbA1c > 14% were recorded as 14.0. Data before and after transition was compared by a paired t-test (pre- and post- transition HbA1c comparison of included subjects), Mann-Whitney-Wilcoxon Test (HbA1c comparison of included versus excluded subjects), two-sided Exact McNemar test (diagnosis of hypertension, treatment with anti-hypertensive medications, diagnosis of dyslipidemia, treatment with lipid-lowering medications, diagnosis of microalbuminuria, treatment with angiotensin-converting enzyme (ACE) inhibitors) and one-sided Exact McNemar test (adherence to lipid screening), and a two-sided Fisher’s Exact test when assessing adherence to microalbuminuria, retinopathy, and peripheral neuropathy screening as the samples were unmatched.

Blood pressure measurements for 1 year prior to transition and 1 year after transition were collected from each diabetes clinic visit. Subjects were characterized as having a diagnosis of hypertension if they had hypertension listed in their medical history or problem list. Subjects were characterized as having undiagnosed hypertension if they met criteria for the diagnosis of hypertension per the ADA ‘Standards of Medical Care in Diabetes’ guidelines [17, 19] but did not have the diagnosis of hypertension listed in their medical history or problem list. For pediatric patients, hypertension was defined by a systolic blood pressure or diastolic blood pressure ≥ 95th percentile for age, sex, and height confirmed on 3 separate diabetes clinic visits prior to transition. Subjects receiving care by an adult provider after transition were characterized as having hypertension if they had blood pressure measurements ≥140/90 confirmed using multiple readings on separate days. Data about treatment with anti-hypertensive medications were recorded for each subject.

Adherence to screening for dyslipidemia was collected on each subject in relation to screening recommendations established by the ADA ‘Standards of Medical Care in Diabetes’ [17, 19]. As such, screening of subjects in the care of a pediatric provider was considered up-to-date if there was a normal lipid panel obtained within the past 5 years prior to transition. However, if there was a history of an elevated LDL level (≥100 mg/dL), screening was considered up-to-date when a lipid panel was repeated annually if the LDL ≥100 mg/dL persisted. Screening of subjects in the care of an adult provider was considered current if there was a normal lipid panel obtained within the past 5 years or annually if the patient was receiving treatment with a statin medication. Subjects that did not meet these screening guidelines, including those who had a total cholesterol level obtained without a full lipid panel, were considered non-adherent to screening guidelines. Subjects were characterized as having a diagnosis of dyslipidemia if they had any lipid disorder (e.g. dyslipidemia, hyperlipidemia, hypertriglyceridemia, pure hypercholesterolemia) listed in their medical history or problem list. Subjects were characterized as having undiagnosed dyslipidemia if their last lipid panel demonstrated an LDL ≥100 mg/dL but did not have the diagnosis of any lipid disorder listed in their medical history or problem list. Data on treatment with statin medications was collected for each subject.

Adherence to screening for microalbuminuria was collected on each subject and determined to be up-to-date if in accordance with guidelines per the ADA ‘Standards of Medical Care in Diabetes’ [18, 19]. Screening of subjects in the care of a pediatric provider was considered up-to-date if a spot urine microalbumin-to-creatinine ratio or 24-h urine creatinine collection was obtained during the 1-year time period prior to transition in patients that had T1D for ≥5 years. Screening of subjects in the care of an adult provider was considered current if there was a spot urine microalbumin-to-creatinine ratio or 24-h urine creatinine collection obtained during the 1-year time period after transition in patients that had T1D for ≥5 years. Subjects with T1D < 5 years were excluded from this analysis, as they were not eligible for chronic kidney disease screening per guidelines. Subjects were characterized as having a diagnosis of microalbuminuria if they had microalbuminuria or other diabetes-related kidney disorder listed in their medical history or problem list. Subjects were characterized as having undiagnosed microalbuminuria if their last urine microalbumin-to-creatinine ratio was ≥30 mg/g but was not recognized and acted upon by their provider or they did not have the diagnosis of any diabetes-related kidney disorder listed in their medical history or problem list. Data on treatment with ACE inhibitors and angiotensin receptor blockers medications was collected for each subject.

Adherence to screening for retinopathy was collected on each subject and determined to be up-to-date if the appropriate exam had been performed as recommended by the ADA ‘Standards of Medical Care in Diabetes’ [18, 19]. Screening of subjects in the care of a pediatric provider was considered up-to-date if there was documentation of an eye exam in the electronic medical record or by subject self-report of the date of their last eye exam performed in the 1-year time period prior to transition, or 2-year time period prior to transition if low risk based on glycemic control, in patients that had T1D for ≥5 years. Screening of subjects in the care of an adult provider was considered current if there was documentation of an eye exam in the electronic medical record or by subject self-report of the date of their last eye exam performed in the 1-year time period after transition, or 2-year time period after transition if low risk based on glycemic control, in patients that had T1D for ≥5 years. Subjects with T1D < 5 years were excluded from this analysis as they were not eligible for retinopathy screening per guidelines. Subjects were characterized as having a diagnosis of retinopathy if they had retinopathy listed in their medical history or problem list. Data on treatment for retinopathy was collected on each subject.

Adherence to screening for peripheral neuropathy was collected on each subject. Subjects were characterized as having up-to-date screening if their diabetes provider had performed the necessary exam and/or tests as indicated by the ADA ‘Standards of Medical Care in Diabetes’ [18, 19]. Each subject’s chart was searched for the terms “neuropathy”, “foot exam”, and “monofilament”, “filament”, “podiatry”, and “podiatrist” to determine if screening was performed. Clinic visit notes during the 1-year time period prior to transition and during the 1-year time period after transition were reviewed for documentation evidence of a foot exam in the physical exam section. Screening of subjects in the care of a pediatric provider was considered up-to-date if a foot exam was performed during the 1-year time period prior to transition in patients that had T1D for ≥5 years. Screening of subjects in the care of an adult provider was considered up-to-date if a foot exam was performed during the 1-year time period after transition in patients that had T1D for ≥5 years. Subjects with T1D < 5 years were excluded from this analysis as they were not eligible for foot exams per guidelines. Subjects were characterized as having a diagnosis of peripheral neuropathy if they had neuropathy listed in their medical history or problem list. Data on treatment with medications for neuropathy was collected for each subject.

## Results

**Fig. 1:**
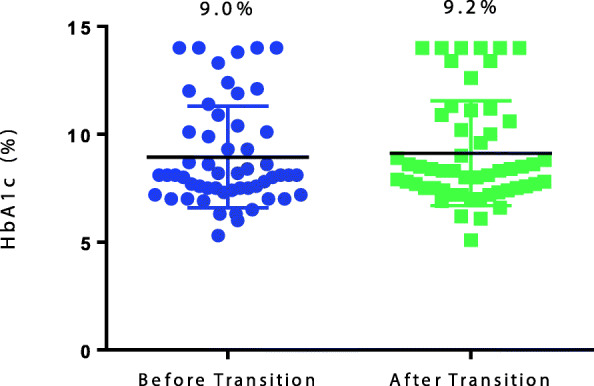
HbA1c before and after transition. HbA1c levels were not significantly different over the transition period when compared by a paired t-test

Of the 397 potentially eligible patients with T1D identified as having been seen in the pediatric diabetes clinic, 68 were excluded as they were still receiving care in the pediatric practice at the time of this chart review, 31 had transitioned to adult providers but < 1 year ago so there was incomplete post-transition data, 100 were “lost to follow up” (i.e., seen only one time in the pediatric clinic, visits ceased without explanation before 18 years of age, moved away from region), and 1 had a restricted record. Of the remaining 197 patients, 54 (27%) were included in the final analysis as they had care transitioned to an adult provider within the academic medical practice and detailed review of medical records both before and after transition was possible. The baseline characteristics of the 54 subjects are included in Table 1 and are thought representative of the institution’s T1D patient population. The mean HbA1c at last visit to the pediatric provider was 9.0% in the 54 patients who remained within MCW and 9.3% in the 143 who transitioned care to non-MCW adult providers (*p* = 0.11). The transition to adult care occurred at a mean age of 18.1 years. The mean time from last visit with a pediatric T1D provider to first visit with an adult T1D provider (i.e. length of transition) was 7.8 months but varied widely from − 3.6 months (patient was seen by an adult provider and then by their pediatric provider once more before ultimately transitioning) to 2.2 years. The HbA1c was stable over the transition period with a mean HbA1c value of 9.0% before and 9.2% after transition (*p* = 0.38, Fig. 1).

**Table 1:** Baseline subject characteristics

	N (%) or Mean (S.D.)
**Gender**	
Male	28 (51.9)
Female	26 (48.1)
**Ethnicity**	
White	42 (77.8)
Black	7 (13.0)
Hispanic or Latino	3 (5.6)
Unknown	2 (3.7)
**BMI (z-score)**	0.58 (± 0.9)
**Age at diagnosis (years)**	8.9 (± 4.4)
**Age at transition (years)**	18.1 (± 1.0)
**Length of transition (months)**	7.8 (± 6.0)

**Fig. 2:**
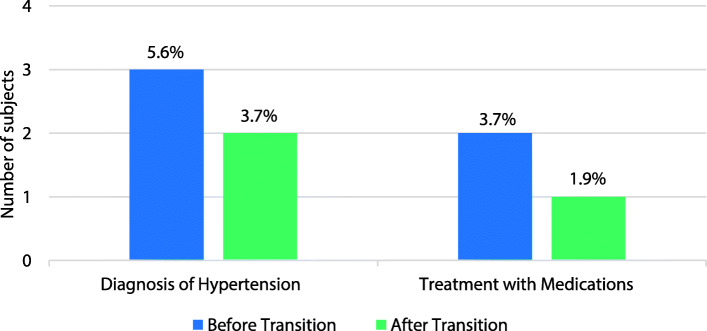
Blood pressure diagnosis and pharmacological treatment. There was no significant difference in diagnoses of hypertension or in the number of patients treated with anti-hypertensives between the groups when compared by a two-sided Exact McNemar test

Hypertension Diagnosis and Management: The number of diagnoses of hypertension (3 versus 2 subjects before and after transition, respectively) and the number of patients treated with anti-hypertensives (2 versus 1 subjects) did not significantly change over the transition period (*p* = 1, Fig. 2). Subjects very rarely met criteria for hypertension per the ADA guidelines, but it was appropriately recognized and documented 100 and 96% of the time before and after transition, respectively.

**Fig. 3:**
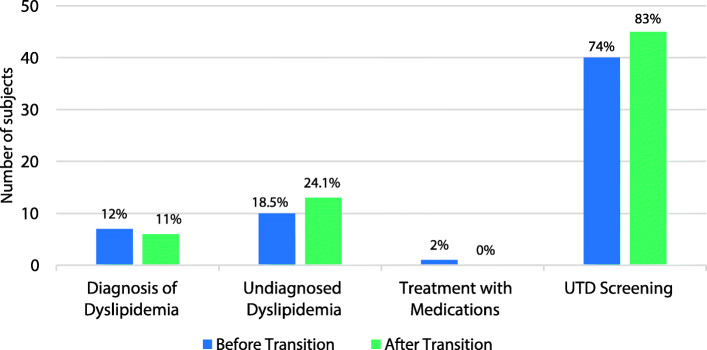
Dyslipidemia diagnosis and management. There was no significant difference in diagnoses of dyslipidemia, undiagnosed dyslipidemia, treatment with statin medications between the groups by a two-sided Exact McNemar test nor was there was a significant difference in adherence to lipid screening between the groups when compared by a one-sided Exact McNemar test. UTD = up-to-date per ADA Standards of Medical Care

Dyslipidemia Screening, Diagnosis, and Management: Subjects received up-to-date lipid screening 74 and 83% of the time before and after transition, respectively, with a trend toward increased adherence rate after transition to adult care that did not reach significance (*p* = 0.13, Fig. 3). Of the 14 and 9 individuals not up-to-date with lipid screening before and after transition, respectively, the need for screening was appropriately recognized and a lipid panel was ordered, but not collected for unknown reasons in 3 subjects before transition and by 1 subject after transition. Before and after transition, 12 and 11% of subjects were appropriately diagnosed with dyslipidemia. However, dyslipidemia remained undiagnosed in 19 and 24% of subjects. More subjects were undiagnosed with dyslipidemia by adult providers but not significantly so. The use of statin medications was overall very low and did not change over the transition period (Fig. 3). The only patient receiving a statin prescribed by a pediatric provider had it discontinued by the adult provider.

**Fig. 4:**
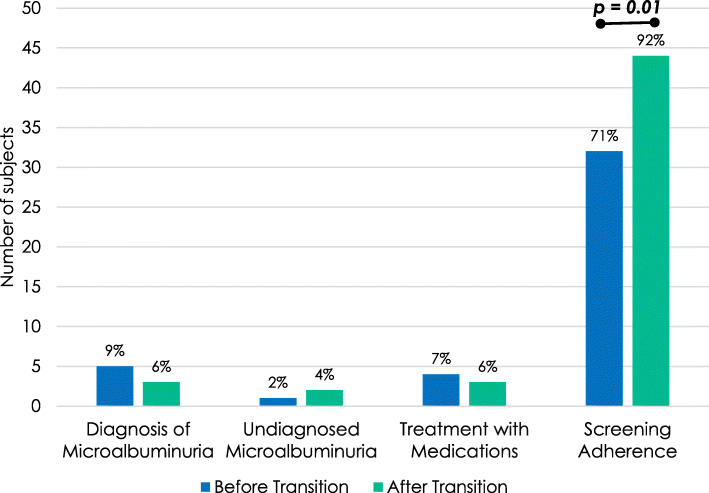
Microalbuminuria diagnosis, management, and screening adherence. There was no significant difference in diagnoses of microalbuminuria or other diabetes-related kidney disease, or treatment with ACE inhibitors between the groups by a two-sided Exact McNemar test. There was a significant difference in adherence to screening for chronic kidney disease between the groups when compared by a two-sided Fisher’s Exact test

Chronic Kidney Disease Screening, Diagnosis, and Management: Screening for microalbuminuria in eligible subjects occurred 71 and 92% of the time before and after transition, respectively, with significantly increased adherence rate after transition to adult care (*p* = 0.01, Fig. 4). Of the 13 and 4 subjects non-adherent to microalbuminuria testing before and after transition, the need for screening was appropriately recognized and ordered by providers but subsequently not collected by 8 subjects before transition and by 1 subject after transition. Screening was performed, although it was not indicated, in 2 subjects before transition and in 5 subjects after transition as they had T1D < 5 years duration. Before and after transition, 9 and 6% of subjects were appropriately diagnosed with microalbuminuria. However, microalbuminuria remained undiagnosed in 2 and 4% of patients before and after transition, respectively, in that there were elevated urine microalbumin-to-creatinine ratios that remained unaddressed by the providers. There were 4 subjects treated with ACE inhibitors by pediatric providers. Of these, 1 had the medication discontinued after transition to adult care when the urine microalbumin-to-creatinine ratio remained within goal off the ACE inhibitor.

Retinopathy Screening: Retinopathy screening on eligible subjects occurred only 80 and 81% of the time before and after transition, respectively. There was no significant difference in rate of adherence to screening by pediatric or adult providers (*p* = 1). Of those not up-to-date with retinopathy screening, the need for screening was appropriately recognized and requested by providers but subsequently not done by 1 subject before transition and by 3 subjects after transition. Screening was performed, although not indicated, in 7 subjects before transition and in 4 subjects after transition as they had T1D less than 3 years. No subjects were diagnosed with retinopathy before transition. One subject did not have an eye exam done while in the care of a pediatric provider despite multiple requests. After transition to an adult provider, an eye exam revealed retinopathy per an optometry report; however, this diagnosis was not added to the patient’s medical history or problem list in the electronic medical record and a repeat eye exam was not done despite being requested.

**Fig. 5:**
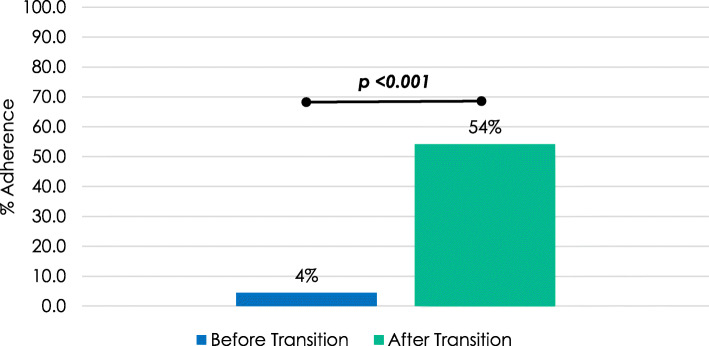
Adherence to neuropathy screening in eligible subjects. Adherence to screening for neuropathy increased significantly after transition when compared by a two-sided Fisher’s Exact test

Neuropathy Screening: Neuropathy screening on eligible subjects occurred less than 5% of the time in pediatric practice (2 of 45 eligible subjects) and only 54% of the time in adult practice (26 of 48 eligible subjects). Despite falling short of guidelines, the adherence to neuropathy screening increased significantly after transition (*p* < 0.001, Fig. 5). There were no subjects with a diagnosis of neuropathy, and no one was receiving medical treatment for neuropathy prior to transition. After transition, one patient received a diagnosis of neuropathy and was subsequently started on gabapentin. This was not statistically significant.

## Discussion

This study demonstrates that in a cohort of young adults with T1D there were no significant changes in the diagnosis or management of hypertension, dyslipidemia, microalbuminuria or neuropathy during the transition period from pediatric to adult care. These findings were counter to the original hypothesis that pediatric providers were inadequately addressing T1D-related complications as compared to adult providers. However, the findings are concerning for the overall suboptimal adherence of both adult and pediatric providers to widely available screening guidelines for hypertension, dyslipidemia, microalbuminuria, retinopathy, and neuropathy.

Adherence to hypertension screening was very high with nearly no missed diagnoses of hypertension, likely due to the routine measurement of blood pressure in clinical practice. Adherence to lipid screening was fair but suboptimal (74–83%); of subjects with up-to-date screening, approximately 20% of subjects had unrecognized or undocumented dyslipidemia before and after transition. Adherence to screening for chronic kidney disease with microalbumin testing increased significantly after transition. Interestingly, screening for chronic kidney disease when it was not indicated also increased after transition suggesting that adult providers may be more apt to reflexively order this screening in patients regardless of guidelines. There was no difference in adherence to retinopathy screening between the two groups; however, it was not performed in approximately 20% of the patients in which screening was indicated. Several subjects had eye exams performed when not indicated, which may reflect eye exams being obtained for reasons unrelated to T1D. Although not significant, the number of patients with undiagnosed hypertension, dyslipidemia, and microalbuminuria did increase after transition. This has important implications for the care of these patients as the presence of these complications should prompt providers to evaluate for possible co-morbid cardiovascular risk factors, discuss necessary lifestyle changes and consider treatment with medications as indicated. Furthermore, given the high concurrent complication burden in patients with T1D [11, 12], providers should be aware of the increased risk of developing further diabetes-associated complications, assess patients regularly according to guidelines, and manage appropriately. Of note, the ADA does not provide firm guidance on indications to rescreen nor an appropriate timeline in which to repeat lipid testing when abnormal in adult patients with T1D, which may potentially create confusion for providers [18, 19]. There has also been increasing uncertainty about extrapolation of cardiovascular risk assessment and pharmacologic intervention from mostly type 2 diabetes to the T1D population [23] as well as increased recognition of adverse effects of statins such as myopathy, which may be particularly bothersome to physically active young adults [24]. These factors may have contributed to the number of “undiagnosed” dyslipidemia and low rate of statin treatment.

Although adherence to screening for neuropathy increased after transition to an adult provider in this study, the rate of adherence with neuropathy screening remained much lower than was anticipated as nearly half of the patients in which screening for neuropathy was indicated were missed. The pediatric providers performed much worse, performing recommended foot exams on only 4% of eligible subjects, highlighting a significant need for practice improvement. Improved adherence to neuropathy screening is most important in young adults as the risk of T1D-associated complications increase with longer duration of disease [11–14], and patients with T1D that develop diabetic foot disease have been shown to have lower self-reported quality of life [25]. These complications may be mitigated if recognized and treated at their onset.

In this study there was no significant change to a given patient’s HbA1c over the transition time counter to what has been shown in prior studies [9]. However, like findings reported by the T1D Exchange [4], the mean HbA1c levels both before and after transition in these young adults were well above the recommended target (HbA1c < 7.5 and < 7% in those less than and greater than 18 years of age, respectively). Prior studies have documented that poor glycemic control is associated with worse health outcomes [10, 11].

There were several limitations of this study. This study was limited to a single academic medical center as this allowed complete access to pediatric and adult clinic electronic medical records. However, this only captured approximately 1/2 of subjects followed in the CW Diabetes Clinic practice who transitioned to adult care at least 1 year prior to the chart review. The other 1/2 of subjects receiving care through the CW Diabetes Clinic transitioned care to outside providers. This reflects the geographically large catchment area for the pediatric diabetes practice and lack of competing non-academic providers in Southeastern Wisconsin as well as the much broader access to adult providers in non-academic groups throughout the same region. As such, the data collected therefore are reflective of a smaller subset of providers under the umbrella of the same institution and may not be generalizable to private practice, for example. Despite this limitation, the subjects studied appear largely representative of the larger pediatric patient population in the CW Diabetes Clinic based on gender, ethnicity, and BMI z-score. Additionally, the HbA1c values were similar between those who remained in the same institution and those who sought adult care elsewhere. An additional limitation is that only blood pressures obtained at diabetes clinic visits were included in the study, and higher blood pressure measurements may have been missed, possibly decreasing the rates of hypertension diagnoses. Not included in this study was collection of data regarding atherosclerotic cardiovascular disease risk factors to determine if patients with diagnosed dyslipidemia would benefit from statin therapy per ADA guidelines. Finally, adherence to neuropathy screening was based on inclusion of specific terms in the medical record or evidence of a foot exam in the physical exam section of clinic notes. Therefore, it is possible that the screening was performed but not documented which could have resulted in a falsely lower rate of adherence.

Our study most significantly highlights the need for practice improvement in the care of patients with T1D. In response to the findings highlighted in this study, the pediatric practice recognized the need for quality improvement and enacted several changes to their practice model. A quality improvement project including a plan-do-study-act cycle has been initiated in attempt to increase rates of adherence to screening for peripheral neuropathy [26]. For example, formal provider education on how and when to perform peripheral neuropathy screening has been conducted, monofilaments have been made available in examination rooms, and a specific diabetic foot exam has been added to the physical examination template in the electronic health record. Rates of adherence to peripheral neuropathy screening will be reviewed 6 months after these interventions to evaluate for improvements. Additionally, given the recognition that the rate of adherence to microalbuminuria screening is low, urine samples are now collected in clinic rather than asking patients to go to a lab for testing.

It is important to note that the ADA ‘Standards of Medical Care in Diabetes’ guidelines are updated annually. This study used the 2018 guidelines when determining adherence to recommended screening parameters. Across the timespan the guidelines have remained largely unchanged when compared to the most recently released guidelines in 2020, particularly in the delivery of care of older adolescents and adults with T1D, which was the population studied in this chart review. However, it is important to note a few of the major changes which have occurred within the past few years. For dyslipidemia screening in pediatric patients, it is now recommended that if the LDL level is within the accepted risk level, a repeat lipid profile should be obtained every 3 years rather than every 5 years as was previously recommended [19, 27]. Retinopathy screening is now recommended in pediatric patients once they are aged ≥11 years or puberty has started, whichever is earlier, and once they have had T1D for 3–5 years [27]. Per the most recent guidelines, retinopathy screening should be performed every 2 years after the initial comprehensive and dilated eye exam but could be performed as infrequently as every 4 years on the advice of an eye care professional and based on risk factor assessment and adequate glycemic control [27]. Previously it was recommended that eye examinations be performed annually with the option to perform every 2 years based on advice of an eye care professional and risk factor assessment [19]. In adult patients, blood pressure targets should now be individualized through a shared decision-making process that addresses risk factors, potential for adverse effects, and patient preferences [28] whereas a previous goal blood pressure of < 140/90 mmHg was recommended for most patients with diabetes and hypertension [17]. Screening for chronic kidney disease should now be performed twice annually in patients with a prior urinary microalbumin-to-creatinine ratio > 30 mg/g and/or an estimated glomerular filtration rate < 60 mL/min/1.73 m^2^ rather than annually as was previously recommended [18, 29]. In order to provide optimal patient care based on the most recent evidence-based guidelines, it is important for providers to stay informed of the screening guidelines which are updated annually.

## Conclusions

There were no significant changes in the diagnosis or management of hypertension, dyslipidemia, chronic kidney disease, retinopathy, or neuropathy during the transition period in a cohort of young adults with T1D. However, the nearly 8-month transition period was longer than the recommended every 3 month outpatient visits for individuals with T1D, highlighting a need for improved collaboration between the pediatric and adult practices. Unlike other studies, this study did not demonstrate a worsening or significant change in glycemic control during the transition period; however, the mean HbA1c values were well above the recommended target both before and after transition. Adherence to chronic kidney disease and neuropathy screening guidelines increased after transition but were not optimal. Perhaps most enlightening was the finding of very poor rates of neuropathy and, to a lesser degree, to chronic kidney disease screening by the pediatric providers. This finding has afforded an opportunity for quality improvement. It is evident that all providers need to practice more vigilance in screening for T1D-related complications, particularly microalbuminuria and peripheral neuropathy, as missed diagnoses are missed opportunities for potential intervention and may portend future complications.

## Data Availability

The data that support the findings of this study are available from the corresponding author upon reasonable request.

## Abbreviations

ACE: Angiotensin-converting enzyme
ADA: American Diabetes Association
CW: Children’s Wisconsin
HbA1c: Hemoglobin A1c
LDL: Low-density lipoproteins
MCW: Medical College of Wisconsin
T1D: Type 1 diabetes
